# Strong coupling of diffraction coupled plasmons and optical waveguide modes in gold stripe-dielectric nanostructures at telecom wavelengths

**DOI:** 10.1038/srep45196

**Published:** 2017-03-24

**Authors:** Philip A. Thomas, Gregory H. Auton, Dmytro Kundys, Alexander N. Grigorenko, Vasyl G. Kravets

**Affiliations:** 1 School of Physics and Astronomy, the University of Manchester, Manchester, M13 9PL, UK; 2 School of Computer Science, the University of Manchester, Manchester, M13 9PL, UK

## Abstract

We propose a hybrid plasmonic device consisting of a planar dielectric waveguide covering a gold nanostripe array fabricated on a gold film and investigate its guiding properties at telecom wavelengths. The fundamental modes of a hybrid device and their dependence on the key geometric parameters are studied. A communication length of 250 μm was achieved for both the TM and TE guided modes at telecom wavelengths. Due to the difference between the TM and TE light propagation associated with the diffractive plasmon excitation, our waveguides provide polarization separation. Our results suggest a practical way of fabricating metal-nanostripes-dielectric waveguides that can be used as essential elements in optoelectronic circuits.

Optical communication is the fastest means of information processing. However, conventional optical components are relatively bulky and cannot be packed together as compactly as the ubiquitous electronic components. Plasmonic nanostructures allow for waveguiding beyond the diffraction limit, making them potential replacements of electronic interconnects in the next generation of CMOS-integrated circuits^1^,^2^. Several types of plasmonic waveguiding structures have been proposed and studied, including metal-insulator-metal and insulator-metal-insulator multilayered structures^3^, strips^4^, V-grooves^5^, wedges^6^, and nanoparticles chains^1^, etc. The possibility of field localization and enhancement suggests that plasmonic waveguides could have a large impact on applications at telecommunication and optical frequencies.

A unique property of these hybrid metallic waveguides is that they can simultaneously carry electronic and optical signals and therefore possess the potential to become electronically tuneable photonic devices, e. g. using graphene as a control element^7^. However, such designs are limited by strong dissipative losses in the constituent plasmonic materials (usually noble metals such as gold or silver)^2^,^5^,^8^,^9^,^10^. Ohmic losses limit the propagation distance of highly-confined surface plasmon polaritons (SPPs) in planar waveguide structures to at best a few tens of micrometers^11^. Although it is possible to create waveguides in which SPPs can propagate for up to a few millimeters^4^,^12^, these long-range SPPs tend to be poorly confined^13^. For example, metal films and strips^1^,^2^,^13^ can guide either long- or short-range SPPs by changing the film thickness, and decreasing the thickness of the film or strip results in poorer localization of the long-range mode.

New waveguide designs have been investigated to overcome this limitation. V-grooves have previously been designed to increase the confined SPP propagation length to around 100 μm^5^. Metal strips and wedges are relatively easy to fabricate but are expected to exhibit relatively large propagation losses and may be sensitive to structural imperfections. A novel hybrid plasmonic waveguide that controls the coupling between dielectric and plasmonic modes has been suggested with high propagation length (approximately 40–150 μm) and field confinement^14^. Hybrid plasmonic waveguides in this case were formed by dielectric nanowires coupled to a metal surface which supports SPPs. The detailed experimental analysis of the actual impact of these factors on plasmon waveguides based on nanostripes is still to be performed. It has also been suggested that losses could be overcome by using gain-enhanced plasmonic metamaterials^8^, replacing the currently used noble metals with other materials such as semiconductors^9^, or by incorporating graphene into the waveguide structure^10^. However, these designs rely on complex fabrication techniques that in some cases have not yet been standardized.

In this work, we experimentally study hybrid plasmonic waveguides in a novel geometry where a dielectric layer is deposited directly onto a regular array of plasmonic nanostripes (NSs) fabricated on the surface of a gold film. This waveguide structure supports tightly-confined hybrid plasmon waveguide (HPWG) modes with long communication lengths (200–300 μm). We investigate the main properties of HPWG modes using angle-resolved optical measurements. The coupling between localized plasmon resonances (LPRs) of the array and guided modes was controlled by tuning the geometrical parameters of gold nanostripes. Rabi splitting of HPWG TM modes was observed.

## Results

### Sample design

**Figure 1:**
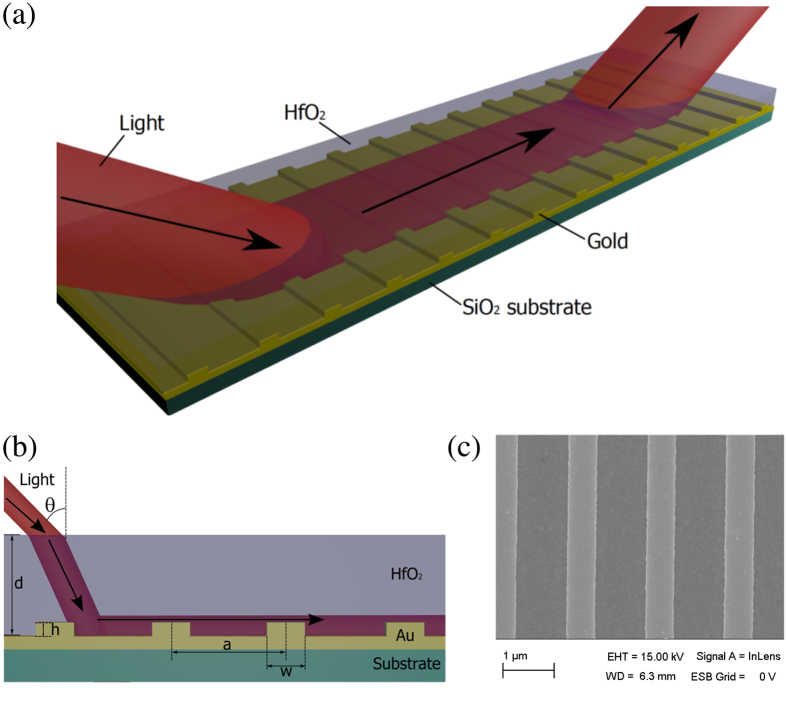
Hybrid plasmonic waveguide devices. (**a**) A schematic illustration of the hybrid structure illuminated by a light with equal incident and scattering angles. (**b**) Schematic illustration of trapping of light (at incident angle *θ*) in NSs-waveguide layer due to scattering on the nanostructure and internal reflection. (**c**) An SEM image of the gold nanostripe sample without the waveguide layer.

Electron-beam lithography was used to fabricate gold nanostripes on top of a flat metallic film. Figure 1(a,b) shows the schematic of the hybrid plasmon-waveguide system. Here, *w* and *h* are the width and height of Au NSs, respectively, *d* is the thickness of the top waveguide layer, and *a* is the period of the NS array. Samples were fabricated using standard electron-beam lithography techniques; see Methods. To choose the optimal height of gold NSs it is possible to simulate our nanostructure using an analytical theory described in ref. 15 and experimentally confirmed for terahertz surface plasmon waveguides in ref. 16. We have investigated the diffraction coupled plasmon resonances in pure gold NSs with fixed stripe wide 400 nm and height in the range from 60 to 90 nm with step of 10 nm. It was found that the stripe height of 70 nm is optimal for achieving the sharpest plasmonic resonances for telecom wavelengths of the best quality: *Q* = *λ*_*res*_/*Δλ* (where *λ*_*res*_ is the spectral position of plasmon resonance and *Δλ* is the full-width at half-maximum)^7^.

The period, *a*, of the line arrays on each sample was fixed around 1500 nm and confirmed by scanning electron microscopy (SEM) after fabrication (we fabricated samples with different *w* and *a*). Figure 1(c) shows a SEM image of the gold NS sample without the waveguide layer. A 400 nm-thick hafnium oxide (HfO_2_) dielectric layer was deposited by electron beam evaporation to cover the NSs and form a waveguide layer on top of the samples. We have chosen HfO_2_ as an optical guiding layer because of its chemical stability and high refractive index of ∼1.9 (at wavelengths of 1300–1550 nm). For HfO_2_, the trapping angle of light can be evaluated as arcsin(1/*n*) ≈ 32° at the air-film interface. This implies that only radiation going into the top 1.1 steradians (which is about 1.1/(4·π) ≈ 8% of the total angle) will partially escape the film into the air. There exists an optimum combination of the gold stripe width, *w*, and their periodicity, *a,* in the dielectric layer (with high real refractive index, *n*) that maximizes the light trapping and optical guiding for such structures. Note that the HfO_2_ layer covers the NSs and forms a waveguide layer on top of the entire sample, which is different from a previously reported metallic photonic crystal slab with a waveguide beneath the NS array^17^. The proposed plasmon-waveguide structure is similar to the structure recently investigated in ref. 18; however, our devices are adjusted for telecom frequencies and can be used as important elements for highly-integrated photonic circuits.

### Sample characterisation *via* spectroscopic ellipsometry

We characterised our samples using variable angle spectroscopic ellipsometry; see Methods. Ellipsometry describes the sample’s optical reflection *via* the ellipsometric parameters Ψ (reflection) and Δ (phase), which are related to the amplitude reflection coefficients for *p*- and *s*-polarized light *r*_*p*_ and *r*_*s*_ by tan(Ψ) exp (*i*Δ) = *r*_*p*_/*r*_*s*_. Note that the ellipsometric function Ψ simultaneously describes plasmon resonances excited by *p*-polarized light (TM modes, which represent dips in spectra, when *r*_*p*_ tends to zero) and TE (*s*-polarized) modes (corresponding to peaks in spectra, when values of *r*_*s*_become very small).

**Figure 2:**
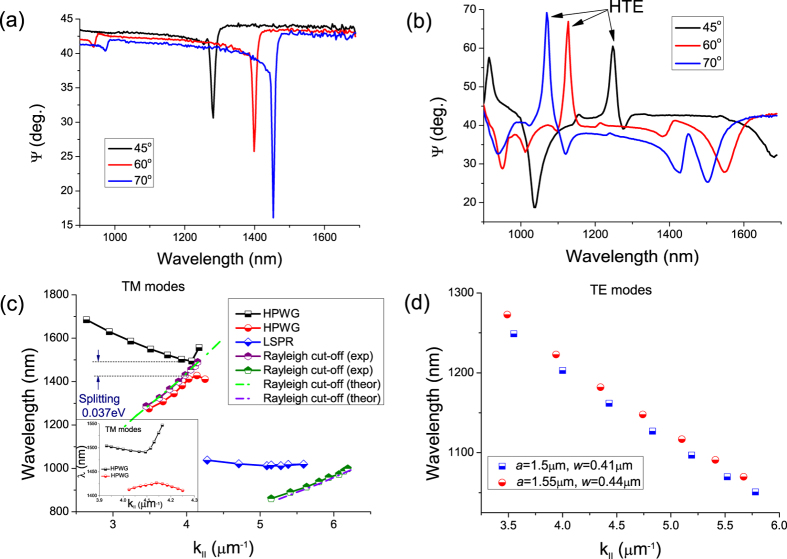
Reflection of hybrid plasmon waveguides and properties of hybrid plasmon waveguide modes. (**a**) Ellipsometric spectra Ψ showing the collective coupled plasmon resonances of pure gold stripes fabricated on a gold layer. (**b**) Ellipsometric spectra of fully fabricated device with identification of HPWG TE ad TM modes. (**c**,**d**) Experimtnal dispersion of guided modes as a function of the wavevector *k*_II_: (**c**) TM modes (Inset demonstrates in detail the dispersion curves near the splitting region); (**d**) TE modes. The data are measured for the sample with *w* = 410, *a* = 1500, *h* = 70 nm and *d* = 400 nm.

The typical high quality plasmonic resonance based on diffraction coupled plasmons measured in our samples without a waveguide layer is shown in Fig. 2(a). The sample has lines *w* = 410 nm wide (with a period *a* ≈ 1500 nm fabricated on a 65 nm gold film.) One can see that a very narrow resonance peak at telecom wavelengths (~1300–1550 nm) was observed as the incident angle *θ* increases from 45° to 70°. Rayleigh-like anomalies in pure plasmonic gold NSs have to be considered as the physical origin for these spectral features^19^. Remarkably, the bandwidth of the resonance at the incident angle of 70° is only ∼8 nm which is approximately 1/200 of the central wavelength 1500 nm. The origin of extremely narrow plasmon resonances in gold stripe arrays fabricated on a gold film is discussed in ref. 7. The drop in the ellipsometric parameter *Ψ* (Fig. 2a) and reflection of *p*-polarized light around the telecom wavelengths were dependent on the angle of incidence. The minimal half-width of the resonant feature observed was just 5 nm (not shown here) which is much smaller than typical values for LPRs. Extremely small widths of resonances in reflection are due to the occurrence of diffraction-coupled localized plasmon resonances^19^ and the drop of light intensity down to zero is due to a strong enhancement of local electric fields near the nanostripes.

Next, we covered the Au NS arrays with a waveguide layer made of HfO_2_. The waveguide layer supports propagating waveguide modes coupled to incident light by the NS array. These modes are often referred to as hybrid plasmon waveguide modes and are quasiguided^17^. They manifest themselves as drops in reflection from the structure. (As far as the ellipsometric parameter Ψ is concerned, hybrid TM modes (*p*-polarization) correspond to dips in Ψ-spectra while hybrid TE modes (*s*-polarization) correspond to the peaks of the spectra). Figure 2(b–d) shows the experimental function Ψ for the same sample with a 400 nm-thick waveguide layer deposited on top. (The fundamental TM and TE HPWG modes are marked by the text.) Compared with the spectral dependence shown in Fig. 2(a), the main resonance wavelength that corresponds to *p*-polarization shifts from 1450 nm (for *θ* = 70°) to 1505 nm, see Fig. 2(b). The minimum at ~1500 nm splits into two dips and a new peak appears at 1075 nm. Interestingly, a peak spectral position for the sample with the waveguide layer demonstrates redshift with increase of the incident angle which is opposite to the blueshift observed for the sample without the waveguide layer, compare Fig. 2(a,b).

In Fig. 2(c) we plot the resonant wavelengths of HPWG TM modes as a function of the wavevector component parallel to the wavevector of the stripe grating, *k*_II_ = *k*_0_sin(*θ*), with *k*_0_ the free space vector and *θ* the angle of incidence. For completeness, we also show the theoretical and experimental dispersion curve of the diffractive-coupled plasmon modes of the bare NS arrays without the HfO_2_ layer. (These modes correspond to the Rayleigh-Wood anomalies^19^,^20^,^21^) The expected position of the Rayleigh cut-off wavelength for air is 
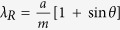
, which gives very good agreement with the experimental data for *m* = 2,3, dash-point lines in Fig. 2(c), measured on NS array before hafnium oxide deposition. Note that the dispersion curve of the Rayleigh cut-off wavelength for *m* = 2 lies exactly between two guided modes denoted by black squares and red circles in Fig. 2(c). Interestingly, the HPWG TM mode exhibits a splitting energy of ∼0.037 eV (Fig. 2(c)). In the case of large *k*_II_ > 4.3 μm^−1^, we observe an almost non-dispersive mode (blue diamonds at Fig. 2(c)) which is most probably connected to the localized out-of-plane resonance of a gold nanostripe. To characterise the coupling efficiency between incident light and plasmonic and waveguide modes, we consider the splitting energy (Fig. 2(b,c)) and the full width of resonance. The splitting was estimated around *E*_*sp*_ ∼0.037 eV (see above) while the linewidth of resonance is smaller than Δ*E*_*res*_ ∼ 0.025 eV (a dip at *λ* ∼ 1500 nm in Fig. 2(b) for *θ* = 70° was taken as an example). Due to fact that the energy splitting is larger than the resonance linewidth, we conclude that the coupling is strong.

In the case of TE modes, gold NSs only function as periodical scatters that do not support LPRs. Figure 2(d) shows the measured spectral dependence of HPWG TE mode. As the periodicity of NSs, *a,* increases from 1500 to 1550 nm, the measured reflection peak shifts toward larger wavelengths (Fig. 2(d)). Finite element modelling confirms the plasmonic origin of the guided modes; see Supplementary Information.

### Communication length of hybrid plasmon-waveguide system

We now describe the most important result of this study. We found that the hybrid plasmon-waveguide system can be used in plasmonic devices that require light transfer over large communication lengths (hundreds of micrometers). The communication length of a waveguide is the distance between the probing input and gathered output signal (see Methods). An analogous conclusion has been reached for a structure of inverted geometry with surface plasmon polaritons excited at a gold surface with a buried grating in the Kretschmann geometry^22^. Figure 1(a) illustrates the schematics of the experiment where light is incident on the device under an oblique angle, propagates along the nanostructure as HPWG mode, and re-emits at the reflection angle. We have measured the spatial distance between the input and output spot of the incident-reflected light by using optical microscope (Methods). It was found that the hybrid plasmon-guide modes can transmit photon energy at the distance of at least 250 μm. This length is larger than the surface plasmon propagation lengths in comparable waveguide designs (which tend to be around 100 μm) and is comparable with the upper limit of the propagation length of surface plasmon polaritons in a flat gold film at 1550 nm (calculated to be 730 μm^22^,^23^).

**Figure 3:**
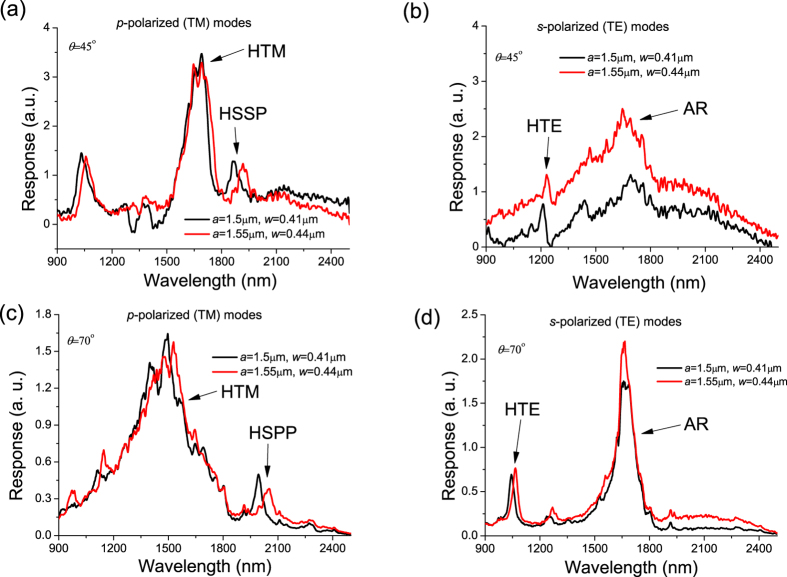
Transfer of light along hybrid plasmonic waveguides. (**a**,**b**) The spectral dependence of the transmitted light intensity inside hybrid plasmonic-waveguide at long distance (∼250 μm) for TM (**a**) and TE (**b**) modes at angle of incidence *θ* = 45°. (**c**,**d**) The same case as (**a**,**b**) but for an angle of incidence *θ* = 70°.

The intensity of transmitted *p*- and *s-* polarized light from waveguide samples at a distance of 200–300 μm from its input was measured at two angles of incidence (45° and 70°) with respect to the separately measured *R*_*p*_ and *R*_*s*_ reflectivity from a flat thick Ag film which provided a background signal. Figure 3 shows the spectral dependence of the intensity of light transmitted at a distance of 250 μm for two values of NSs periodicity, *a*, for *p-* and *s-* polarized light when angle of incidence is equal to 45° (smaller than the Brewster angle, *θ*_*B*_ ≈ 61°, for HfO_2_) and 70° (larger than the Brewster angle for HfO_2_). The light transmitted along the waveguide modes correspond to peaks in measured transmission. For clarity, we labelled the transmitted modes, see the Discussion section. This yields a length for the propagation of HPWG modes on the order of hundreds of micrometres, which suggests a strong contribution from coupling between plasmon and waveguide modes. While the detailed mode analysis of the fabricated plasmon-waveguide devices and their waveguiding characteristics and the excitation efficiency is still to be carried out, the fact of radiation transfer between the in- and out-coupling gold stripes is clearly seen from Fig. 3. In addition, the position of propagated modes can be tuned in the plasmon-waveguide device by changing the angle of incidence of pumping light. The excitation conditions for HPWG TE and TM modes are different which implies that the structures can also function as a polarization sensitive device.

## Discussion

A flat dielectric layer of HfO_2_ deposited on a gold film possesses propagating waveguide modes. In the presence of a nanostripe array fabricated on a gold film, these waveguide modes can couple to light continuum outside the waveguide and become leaky and lossy. The coupling happens due to re-emission of light by the nanostripes (diffraction coupling with array wavevector mismatch). This coupling is most effective for wavelengths close to the localised plasmon resonance of a nanostripe. As a result, LPR resonances in the studied structure can be important for device operation since they define both coupling strength and the mode loss coefficient.

**Figure 4:**
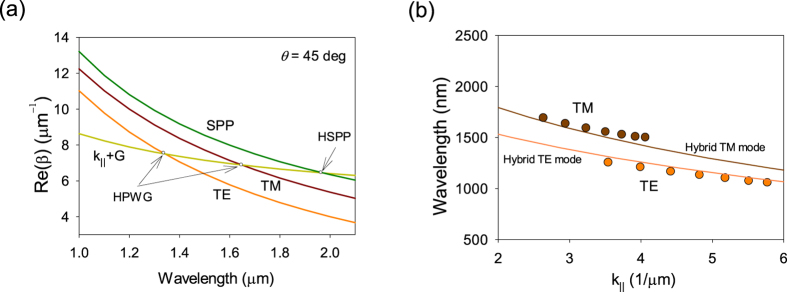
Hybrid plasmon modes of the structure. (**a**) The dispersion of TE and TM modes of a flat dielectric waveguide (air-dielectric-metal) calculated for angle of incidence *θ* = 45°, maroon and brown curves, respectively, combined with dispersion of SPP mode (dielectric-metal) and the light line with momentum mismatch. (**b**) Comparison of calculated dispersion of HPWG TE and TM modes with experimental data plotted in Fig. 2(c,d).

In the first approximation, we model our device as a metal-dielectric-air multilayer system. In such a system the dispersion relation for TM and TE-guided modes can be found from the equations^24^,^25^


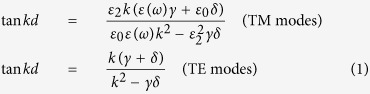


where *ε*_0_, *ε*_2_, and *ε(ω*) are the dielectric constants for air, HfO_2_ and gold (see Supplementary Information). The parameters *k, γ,* and *δ* are given by refs 24 and 25: 
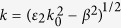
, 
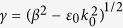
, and 
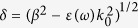
, where *β* is the wavevector of the guided modes and *k*_0_ = 2*π/λ* is the wavevector of photons in air. Figure 4(a) plots the spectral dependence of the wavevector of fundamental (lowest energy) TE and TM modes calculated with the help of (1). These wavevectors are large and cannot be produced by external light. However, in the presence of a periodic NS array, the momentum mismatch can be overcome by the reciprocal vector of the array *G* = 2*π/a*. Figure 4(a) shows that the line *k*_||_ + *G* (plotted for the angle of incidence *θ* = 45° as an example) indeed intersects the TE and TM mode lines at wavelengths 1340 nm and 1650 nm respectively, which correspond to excitation conditions of hybrid plasmon-waveguide modes (the intersections are shown by open circles). These theoretical values are close to the experimentally observed HPWG TE and TM modes in both reflection (at wavelengths of 1250 nm for HPWG TE mode and 1680 nm for TM mode, see Fig. 2(b)) and light transmitted along the waveguide (1210 nm for HPWG TE mode and 1690 nm for TE mode, Fig. 3(a,b)). By changing the angle of incidence we can find spectral behaviour of HPWG modes and compare it with the measured data, see Fig. 3(b). It is clear that calculated fundamental HPWG TE and TM modes (maroon and brown lines) are in reasonably good agreement with the measured positions of HPWG TE and TM modes (maroon and brown circles).

In addition to HPWG modes, our structures possess other plasmon modes. First, surface plasmon polaritons can be excited at the metal interface. A SPP mode on a flat gold film is described by the following spectral relation


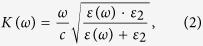


where *K(ω*) is the propagating vector of the SPP mode. Figure 4(a) plots the SPP mode (green line). As in case of waveguide TE and TM modes, SPP mode can only be excited by external light due to the presence of the periodic NS array. The intersection of the line *k*_||_ + *G* with SPP line gives the position of the hybrid surface plasmon polariton mode (HSPP) at wavelength 1970 nm which is reasonably close to the measured position of HSPP (at wavelength of 1860 nm, see Fig. 3(a)). By comparing the amount of light transmitted by HSPP and HPWG modes, we can conclude that HPWG modes transfer about 4 times more light than HSPP at the communication distance of 250 μm. Second, localised out-of-plane plasmons (excited in *p*-polarization) can re-scatter light to reach the detector. These plasmons result in a weakly dispersive mode seen in Fig. 2(c). Third, in addition to HPWG TE modes in s-polarization we observe an “anomalous resonance” (AR) at around 1750 nm which is vaguely independent of angle of incidence, see Fig. 3(b,d). The origin of this feature requires further investigation and will be discussed in future work.

It is interesting to note that the band gap observed for the fundamental TM mode (*p*-polarization) is opened at the propagating wavevector *k*_||_ ≈ *G*, which is different from the standard Bragg condition *k*_||_ = *G*/2 and corresponds to higher order Bragg matching. Near the bandgap position, one can expect slow group velocity of the light. The group velocity of propagating light modes can be found as *v*_*g*_ = d*ω*/d*β*. It corresponds to the group effective refractive index *n*_*g*_ = *c/v*_*g*_ = d*β*/d*ω*. In case of our structures, *β* = *k*_||_ + *G* and the corresponding group index can be approximately evaluated from the relation *n*_*g*_ = *c* · Δ*k*_||_/Δ*ω* which yields *n*_*g*_ ≈ 10 from data plotted in the Inset of Fig. 2(c). We conclude this section by noting that HPWG modes (and others) require a more advanced treatment which would take into account an effective layer produced by the periodic array of NS.

## Conclusion

Hybrid metal-dielectric waveguides have been shown to transfer light with relatively low losses and large communication distances (hundreds of micrometres) at telecommunication wavelengths. Based on photonic waveguide theory, the location and bandwidth of the hybrid plasmonic-waveguide resonances can be tuned by the geometrical parameters of the gold NSs and the angle of incidence of light. As a result, metal-nanostripes-dielectric waveguides could be applied to process information in optoelectronic circuits.

## Methods

### Sample fabrication

First, a 3 nm-thick Cr adhesion layer and 65 nm Au film were deposited on top of a 1 mm-thick glass substrate using electron beam evaporation. Then, the sample was spin coated with a bilayer PMMA resist for electron beam lithography. Second, 300 μm arrays of 100 μm long, *w* = 400–450 nm wide gold stripes with a fixed height *h* = 70 nm were fabricated atop this flat gold region. A 400 nm-thick hafnium oxide (HfO_2_) dielectric layer was deposited by electron beam evaporation to cover the NSs.

### Optical measurements

To characterise the optical properties of the samples we used a variable angle Woollam M2000 F focused beam spectroscopic ellipsometer. The spot size on the sample was approximately 30 μm × 70 μm at ~60° angle of incidence. Ellipsometry measures the ratio of the reflection coefficients making it a very sensitive measurement technique.

The experiments to determine communication length for both the TM and TE hybrid guided modes have been carried out using our confocal microscope with the ability to accurately excite and collect the light at various spatial positions on the sample. The experimental setup consisted of a laser driven broadband light source, Glan-Thompson polariser on a rotating stage, focusing optics (x20 super long working distance objective, working distance 25 mm). We have used multimode broadband fibres with core diameter of 20 μm to deliver and collect the light signal, which acted as a spatial filter. The plasmon-waveguide structure was illuminated on the left edge (see Fig. 1(a)) with a broad spectrum light at angles of incidence 45° and 70° by using an objective lens to give a spot size less than ∼30 μm. To image the focused light spot we have used both an 8% reflectivity beam splitter and a CCD camera in a standard microPL arrangement or high zoom, high magnification CCD camera mounted at a normal incidence to the sample. A small fraction of the excited light was backscattered by the sample grating enabling precise monitoring and positioning of the focused spot. The sample was mounted on a 3D micro-positioning stage. Before each measurement (i.e. 45° and 70°) the sample was replaced by a silver mirror in order to take a reference of the reflected light in each polarisation. In order to define our out-coupling position in space (the other end of the communication length) we have used confocal reverse light coupling. This is achieved when the light is coupled from the collection fibre port and is focused by a collection lens forming a spot on the other end of the sample. The position of this spot was carefully adjusted by moving the XYZ translation of the collection lens. The collection spot could also be viewed by the CCD cameras. Once the alignment of both focusing and collection points was completed the light was again coupled from the input fibre port. The collection fibre was sent to a NIRQuest spectrometer to analyse the light output collected only from a predefined spot on the sample. The spatial distance between the input and output spot of the incident-reflected light was estimated using an optical microscope. In our experiment, we have measured the spectral dependence of the transmitted light at long spatially distributed distance of 250 μm for our plasmon-waveguide structure (Fig. 1). To check the optical setup, we have measured transmitted light intensity in same geometry as shown in Fig. 1(a,b) for pure gold stripes and gold stripes with 70 nm-thick HfO_2_ film for the same communication length. In both cases intensity of transmitted light was at level of standard noise (measurements not shown here).

## Additional Information

**How to cite this article:** Thomas, P. A. *et al*. Strong coupling of diffraction coupled plasmons and optical waveguide modes in gold stripe-dielectric nanostructures at telecom wavelengths. *Sci. Rep.* **7**, 45196; doi: 10.1038/srep45196 (2017).

**Publisher's note:** Springer Nature remains neutral with regard to jurisdictional claims in published maps and institutional affiliations.

## Supplementary Material

Supplementary Information
