# Supplementary material for: Strong coupling of diffraction coupled plasmons and optical waveguide modes in gold stripe-dielectric nanostructures at telecom wavelengths
Source: Sci Rep. 2017 Mar 24;7:45196. doi: 10.1038/srep45196 (PMC5364552; doi:10.1038/srep45196)
Supplement: Supplementary Information [file srep45196-s1.pdf]

Supplementary information for

Strong coupling of diffraction coupled plasmons and optical waveguide modes in gold stripe-dielectric nanostructures at telecom wavelengths

Philip A. Thomas[1]*, Gregory H. Auton[2], Dmytro Kundys[1], Alexander N. Grigorenko[1], Vasyl G. Kravets[1]

[1]*School of Physics and Astronomy, the University of Manchester, Manchester, M13 9PL, UK*

[2]*School of Computer Science, the University of Manchester, Manchester, M13 9PL, UK*

*philip.thomas-2@manchester.ac.uk

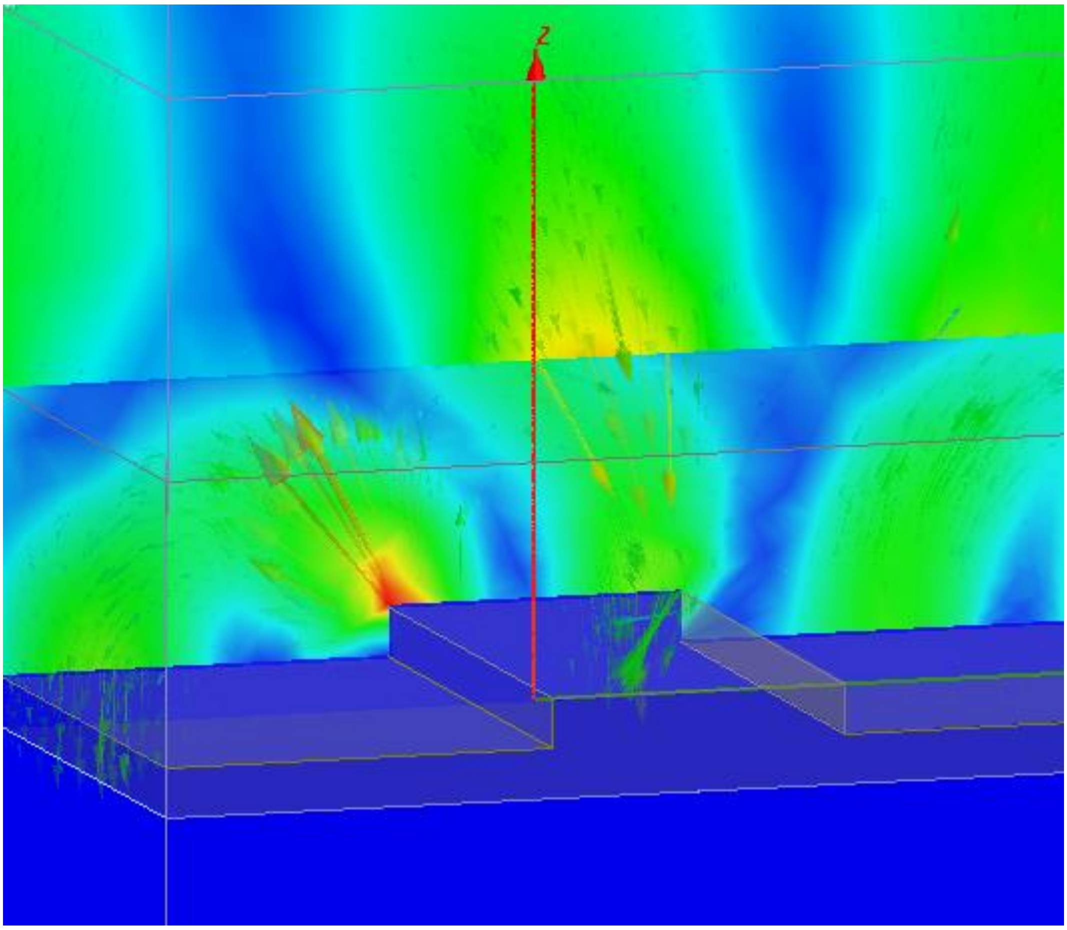

Figure S1 | Finite element simulation of electric fields in hybrid air-dielectric-metal strip waveguide. Finite-element simulations were carried out using HFSS for light incident on the waveguide at 70° with periodic boundary conditions. The simulated fields clearly show coupling of the fields at the corner of the gold nanostripe associated with localised surface plasmon resonances with the fields corresponding to propagating modes away from the gold nanostripe. Simulations also showed this device has a plasmon resonance wavelength at ~1.5 μm (graph not shown), agreeing with out experimental observations in Figs. 2-3.

Dielectric function of gold

In our calculations of the dispersion for TM and TE guided modes (Eq. 1 in the main text) we assume that the permittivity of Au nanostripes of thickness ~70 nm is the same as that of a gold film of thickness ~70 nm. To find the optical constants, we have evaporated a thin gold film under the same conditions that were used in technology processes for preparing of Au NSs. We have exacted the spectral dependences of the complex refractive index $n + ik = \sqrt{\varepsilon(\omega)}$ for the fabricated gold films using spectroscopic ellipsometry (performed with a Woollam spectroscopic ellipsometer) [1]. The extracted dielectric function, $\varepsilon(\omega)$, was then modelled by fitting with three Lorentz functions and the Drude term

$$\varepsilon(\omega) = \varepsilon_0 - \frac{\omega_p^2}{\omega^2 + i\omega\gamma} - \sum_{j=1}^{3} \frac{\Delta\varepsilon_j \Omega_j^2}{\omega^2 - \Omega_j^2 + i\omega\Gamma_j} \qquad (1)$$

with fitting parameters of: ε_0 = 3.9; $\Delta\varepsilon_j$ = (0.25,0.5,3.75); $\hbar\omega_p$ = 8.97 eV; $\hbar\gamma$ = 0.03 eV; $\hbar\Omega_j$ = (2.68,3.09,4.35) eV; $\hbar\Gamma_j$ = (0.449,0.845,3) eV, where j = 1,2,3 [2]. The optical constants of an HfO_2 film have been parameterized by the Cauchy function: $n_d(\lambda) = A_n + B_n / \lambda^2 + C_n / \lambda^4$ (where Cauchy's coefficients are: A_n=1.9, B_n=0.0189, C_n=0.0004 and λ(μm)). To find Cauchy coefficients we again carried out spectroscopic ellipsometry measurements of a pure HfO_2 film. The refractive index of HfO_2 film shows dispersion throughout the visible and near IR spectral range.

References

1. Azzam, R. M. A. and Bashara, N. M. *Ellipsometry and Polarized Light.* (North Holland Press, Amsterdam, 1987).

2. Kravets, V. G., Schedin, F. and Grigorenko, A. N. "Plasmonic blackbody: Almost complete absorption of light in nanostructured metallic coatings." *Phys. Rev. B*, **78,** 205405 (2008).
